# Conditions for Social Exclusion Leading to Distress Change in Chinese Lesbian, Gay, and Bisexual (LGB) People

**DOI:** 10.3390/ijerph20105911

**Published:** 2023-05-22

**Authors:** Chau-kiu Cheung, Eileen Yuk-ha Tsang

**Affiliations:** Department of Social and Behavioural Sciences, City University of Hong Kong, Hong Kong 518000, China

**Keywords:** distress, LGB, social exclusion, stress–vulnerability, Chinese

## Abstract

Lesbian, gay, and bisexual (LGB) people are likely to be at risk of distress because of social exclusion, including the feelings of resentment, resistance, and rejection they might experience from society. Nevertheless, the conditions for social exclusion leading to changes in distress are empirically unclear, especially in Chinese LGB people. To examine these conditions, this study surveyed 303 Chinese LGB people in Taiwan, Hong Kong, and various places in Mainland China. For comparability with other LGB studies, the study did not explicitly identify asexual, demisexual, or pansexual people in the LGB group. Results show that the retrospective reporting of social exclusion in 2016 did not significantly and unconditionally predict levels of distress in 2017. However, the reporting of exclusion significantly predicted current distress when the retrospective report of distress in 2016 was high. These results from the stress–vulnerability model indicate that prior distress is a vulnerability condition that allows social exclusion to exert its stressful effect. This study implies the need to prevent the social exclusion of highly distressed LGB people.

## 1. Introduction

Lesbian, gay, and bisexual (LGB) people (possibly including those alternatively identifying themselves as pansexual rather than restrictively homosexual or bisexual [1]) have a higher risk for distress or emotional problems than others [2]. This risk may be due to social exclusion, including the resentment, resistance, rejection, and discrimination of society towards LGB people as a target group [3]. Nevertheless, given recent findings, the effects of social exclusion on distress have not been found to be uniformly consistent [4,5]. Hence, a stress model regarding the contribution of social exclusion to distress is likely insufficient [6,7]. Instead, stressful effects are likely to be contingent on some conditions. This conditioning means that combinations of some factors may generate interaction effects rather than the main effects of the factors taken individually. According to the stress–vulnerability model, a condition determining the effect of experienced stress is a person’s vulnerability [8,9]. Thus, a possible vulnerability condition is an LGB person’s prior distress, while other LGB people might be less vulnerable. As such, an LGB person’s prior distress is likely to sustain the stressful effect of social exclusion on later distress. This study thus aims to examine the stress–vulnerability model by analyzing survey data obtained from Chinese LGB people to examine the main and conditional effects of social exclusion.

Chinese LGB people, including those in Mainland China, Taiwan, and Hong Kong, are numerous and significant but rarely examined [10,11,12,13,14,15,16,17]. With an assumption that LGB people represent 1% of the Chinese population, the Chinese LGB population can be said to be 0.14 billion [18]. Knowledge about this population is necessary to supplement that which has been primarily obtained in the Western world. The supplement is justifiable in the distinctive collectivist Chinese context [16]. This context foregrounds the risk of social exclusion for LGB people, as well as other Chinese people. LGB people have experienced discrimination in China, and societal and institutional prejudice, stigmatization, and maltreatment against LGB people are prevalent. Accordingly, these practices tend to associate LGB sexuality with problems and shame; discouraging and scapegoating LGB people because of the Chinese emphasis on familism and procreation. This context, meanwhile, tends to urge LGB people to integrate with heteronormative society and excludes LGB people that fail to do so. Nevertheless, this context does not embrace the fundamentalist religious regard of LGB sexuality as sinful [16]. Hence, the extent and ways that social exclusion changes distress in LGB people are uncertain and need scrutiny and possible contextual intervention. At the very least, contextual intervention in the West can upgrade LGB people’s well-being [19].

Distress is a frustrating problem for LGB people, one that requires relief and related research [2]. In the Chinese context, LGB people’s distress has been prevalent, as it involves conformity to heteronormativity and concealment of sexual orientation [20]. Distress is a problem because it provokes other problems, including sexual dysfunction and suicide [21,22]. Additionally, distress is problematic because it impedes health [23] and thus necessitates the need for help. In response to this need, many interventions, treatments, and other practices have emerged to tackle this distress [24]. Nevertheless, the effects of practical treatments and other factors on distress in LGB people are not very certain and thus need further research [25].

The social exclusion of LGB people by society, through rejection, resistance, and discrimination, is prevalent [26]. In the Chinese context, social exclusion can involve censorship in the media, as well as homophobia, marginalization, shaming, and stigmatization [16,27,28,29]. Such exclusion can generate depression, psychopathology, and suicide, as well as distress [30,31,32,33,34]. Additionally, social exclusion can impede health, achievement, and social integration [35,36,37]. Apart from the harm that it inflicts, social exclusion is controversial because it violates the norms of fairness and equality [38]. Social exclusion can further polarize people in society, aggravating and perpetuating societal deprivation [39,40]. Hence, social exclusion is a social pain that urgently needs to be addressed through policy and practice [41,42]. One relevant policy measure is to encourage and facilitate social participation and integration [43].

### 1.1. Expected Effects on Distress

The stress–vulnerability model is likely to explain the main and interaction effects of stress and vulnerability on distress [8,9]. Stress refers to the taxing experiences of LGB people due to their sexual orientation, including social exclusion, rejection, discrimination, stigmatization, or violence against them [30,44]. Subsequently, stress can invoke coping mechanisms; however, when these mechanisms fail, distress is generated [44,45]. The effects can rest on the internalization of this stress, resulting in internalized homophobia in the case of homosexuality [30,44]. Such stress has been responsible for depression, psychopathology, and suicide, as well as distress [32,33,34,44]. Meanwhile, vulnerability refers to a pre-existing weakness in a person that underlies existing problems and the way that they are exacerbated due to stress [9,46]. In their aggravation of the deleterious effect of stress, vulnerability might include a person’s depression, their identification with homosexuality, and their experience of victimization [33,47,48]. Notably, vulnerability also includes prior distress, predisposition to later distress and illness [49,50]. Prior or chronic distress may also aggravate the noxious effect of stress [51,52].

As a form of stress, social exclusion is likely to engender distress. Notably, social exclusion in terms of discrimination, stigmatization, and the experience of violence has raised distress [13,30,37,53]. These social exclusion effects are consistent with other effects due to stress, including internalized homophobia, restrictions, prejudice, and the experience of trauma [54,55]. Conversely, social inclusion in the Western context has lessened LGB peoples’ distress [19]. In the Chinese context, the distressing effect of social exclusion can arise from collectivist pressure on conformity to social norms that maintain social cohesion [20,44].

As the stress–vulnerability model suggests, social exclusion is also likely to have a greater effect on distress when prior distress is higher. Accordingly, the aggravation reflects the multiplication of stress and vulnerability regarding social exclusion and prior distress, respectively [53]. Such aggravation has happened alternatively in interactions involving stress, depression, experience of racism, and social exclusion [48,53]. In the Chinese context, such vulnerability to the distressing effect of social exclusion can rest on shame and the collectivist orientation [16,20].

### 1.2. Hypotheses

The stress–vulnerability model envisions the following hypotheses about Chinese LGB people.

1. Social exclusion is positively predictive of later distress.
2. Social exclusion is more positively predictive of later distress when prior distress is higher.

These hypotheses need testing because of ambivalent or contradictory evidence. Such evidence suggests that generalized stress and stress regarding the experience of abuse, unfair treatment, and internalized homophobia may not breed distress [30,56].

To distill the net effects for hypothesis testing, controlling for prior distress and background and for the response characteristics that might potentially confound the effects is necessary. Prior distress has clearly been a distress predictor [44]. Moreover, distress can breed social exclusion [57]. The background characteristics include the type of LGB (homosexual or bisexual), gender, age, education, residence (rural or urban), and location (Mainland China, Taiwan, or Hong Kong). A person’s distress is typically higher if they are bisexual as opposed to homosexual, female as opposed to male, younger, less educated, or live in an urban as opposed to a rural region [30,45,58]. The experience of social exclusion is more prominent for a homosexual man than a homosexual woman [41]. Acquiescence and social desirability are response qualities or methodological artifacts that require regulation [59,60]. Acquiescence means the tendency to rate everything highly, and social desirability means the tendency to respond in a socially desirable way. Notably, social desirability is potentially confounding, although it has been lower in LGB people than in others [61]. All of these characteristics may affect the outcomes of distress and the experience of social exclusion and thus confound relationships among the outcomes.

## 2. Method

### 2.1. Participants

The study recruited 303 Chinese LGB adults through organizations composed of or concerned with LGB people to participate in and provide valid responses to a self-administered web survey in 2017. This recruitment criterion required the respondents’ affiliation with the organizations to affirm their LGB status in the study sites. As such, the organizations identified the respondents in order to ensure their identities so that both the organizations and respondents could receive honoraria for the survey. Such organizational affiliation was necessary to locate Chinese LGB people who are typically hidden [16]. These respondents were not intended to represent the population because there is no available representative sampling frame. The web survey was particularly suitable for this sensitive topic and participants [62]. Its self-administration circumvented any interviewer bias. Initially, a planned sample of 300 met the need to test at least a weak effect (|*r*| > 0.126) with 95% confidence and 70% statistical power.

These organizations had contacts with LGB people in Taiwan, Hong Kong, and various parts of Mainland China. Eventually, among the participants, 178 were found in Hong Kong, 18 in Taiwan, 30 in Tianjin, 20 in Beijing, 4 in Shanghai, 1 in Shenzhen, and 52 in other places in Mainland China. In order to represent Chinese LGB people, the study assigned different weights to participants in different places to get a weighted sample [62]. The weight was proportional to the population size ratio over the sample size in the location. Hence, participants in Mainland China had greater weights than those in Hong Kong or Taiwan.

The weighted sample showed that 36.8% were female, 75.7% were homosexual, 24.3% were bisexual, and 25.2% were in rural areas. These LGB people had an average of 23.5 years in age (*SD* = 4.5) and 15.8 years in education level (*SD* = 2.9). Because of the variations of the characteristics across sites (see Table 1 and Table 2), hypothesis testing was needed to control for the characteristics.

### 2.2. Measurement

The survey questionnaire interspersed rating items to measure distress, prior distress, and experiences of social exclusion (see Table 1). Each item took a five-step scale to generate scores on a 0–100 scale, with a score of 0 for the first step, 25 for the second step, 50 for the third step, 75 for the fourth step, and 100 for the fifth step. This linear transformation enhanced the ease with which we could interpret and compare the scores without distortion [63]. Some items employed negativelyphrasing, which required the reversion of scoring to check and minimize acquiescent responses [64]. The items also allowed for the identification of and thus control for the acquiescent method factor to distill trait factors independent of the method factor [59,60].

Distress in 2017 combined seven items, such as experiences of “feeling nervous” and “feeling flurried” during the previous fortnight [65]. Based on confirmatory factor analysis, this showed a composite reliability coefficient of 0.899 [66].

Prior distress in 2016 combined seven items, such as experiences of “feeling nervous” and “feeling flurried” in 2016 [65]. Based on confirmatory factor analysis, this showed a composite reliability coefficient of 0.870 [66].

Social exclusion experienced in 2016 combined four items, such as experiences of “society rejecting you” and “society resisting you” in 2016 [67]. Based on confirmatory factor analysis, this showed a composite reliability coefficient of 0.934 [66].

Social desirability in 2016 combined three items, such as experiences of “being ready to help others” and “being confident in your judgment” in 2016 [68]. Based on confirmatory factor analysis, this showed a composite reliability coefficient of 0.842 [66].

Acquiescence was the average of all rating items to represent the tendency to rate every item highly. It was a control factor used in statistical analysis. 

### 2.3. Analysis

Statistical analysis of the weighted sample data proceeded with confirmatory factor analysis to verify the factorial validity of the measurement for regression analysis to test the hypotheses (via M*plus*, [69]). The confirmatory factor analysis identified five trait factors: distress in 2017, distress in 2016, social exclusion experienced, and social desirability, along with a method factor of acquiescence. This analysis verified the factorial or structural validity, comprising the convergent and discriminant validity of the trait factors of distress, social exclusion, and social desirability [70]. Each item was loaded on a respective trait factor and a method factor to maintain discriminant validity [59]. Meanwhile, loadings on the trait factors represented convergent validity. Convergent validity and discriminant validity together indicated factorial validity. With the validity of the measurement, regression analysis then held distress in 2017 as the outcome and prior distress, experience of social exclusion, their interactions, and background and response characteristics as predictors. To minimize the problem of multicollinearity, the interaction was the product of prior distress and social exclusion experienced in terms of their standard scores [71]. This computation also applied to the interaction between gender and sexual orientation as an additional control factor. That is, a lesbian’s distress was seen to be higher than that of a gay or bisexual person. The regression analysis proceeded in two steps to highlight the main and additional interaction effects. Nevertheless, analysis of the main and interaction effects was subject to the assumption of effect linearity [72,73]. Essentially, the analysis revealed changes in distress due to each predictor by controlling for prior distress.

## 3. Results

The average Chinese LGB person displayed modest levels of distress in 2017 and 2016 (*M* = 43.9 and 45.2 and *SD* = 17.2 and 16.4, on a 0–100 scale). Meanwhile, the average LGB person also had modest levels of experienced social exclusion in 2016 (*M* = 42.9 and *SD* = 24.1, on a 0–100 scale). Additionally, the average LGB person held a moderate level of social desirability (*M* = 57.3 and *SD* = 20.3, on a 0–100 scale). Nevertheless, social desirability did not significantly predict distress, as shown later. 

The measures exhibited factorial validity based on the confirmatory factor analysis. Accordingly, they displayed convergent validity in their substantial loadings (0.434–0.816 on distress in 2017, 0.480–0.665 on distress in 2016, 0.595–0.909 on social exclusion experienced, and 0.439–0.820 on social desirability; see Table 3). Their discriminant validity emerged from their separation of the different trait and method factors. Eventually, the trait factors were distinguishable, even with significant correlations among themselves, after controlling for the acquiescent method factor (see Table 4). The confirmatory factor analysis was adequate, given its good fit (*L^2^*(325) = 1906, *SRMR* = 0.064, *RMSEA* = 0.028, and *CFI* = 0.966; [74]).

The first step of the regression analysis did not support Hypothesis 1 about the increase in distress due to social exclusion. Herein, the effect of social exclusion experienced in 2016 on distress in 2017 was nonsignificant (*β* = 0.009, see Column (1) in Table 5), given the control for prior distress and background and response characteristics. Essentially, the effect was credible when the analysis had no problem with multicollinearity (*tolerance* = 0.746–0.942). Notably, the correlation between distress in 2017 and social exclusion experienced in 2016 was significantly positive (*partial r* = 0.149, see Table 4), with the control for acquiescence only. These findings indicate that experience of social exclusion correlated with distress but did not have a net effect on distress after the control for prior distress and other characteristics. In other words, the correlation was attributable to common relationships with prior distress and other factors. Ultimately, social exclusion did not generate an additional increment in distress.

**Figure 1:**
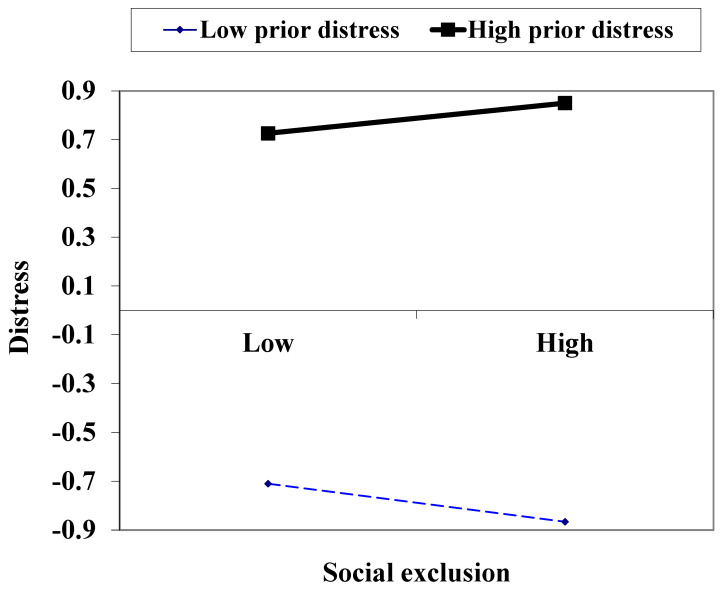
Standard score of distress by high (1 *SD* above *M*) and low levels (1 *SD* below *M*) of experienced social exclusion and prior distress.

The second step of the regression analysis also supported Hypothesis 2 about the greater effect of social exclusion when prior distress is higher. Herein, the interaction effects of social exclusion and distress in 2016 on distress in 2017 were significantly positive (*β* = 0.064, see Column (2) in Table 5), with the control for other predictors. That is, the effect of social exclusion was more positive when prior distress was higher. Notably, social exclusion raised distress only when prior distress had been high (see Figure 1). The interaction or conditional effect was credible when the analysis displayed no multicollinearity problem (*tolerance* = 0.455–0.933).

**Figure 2:**
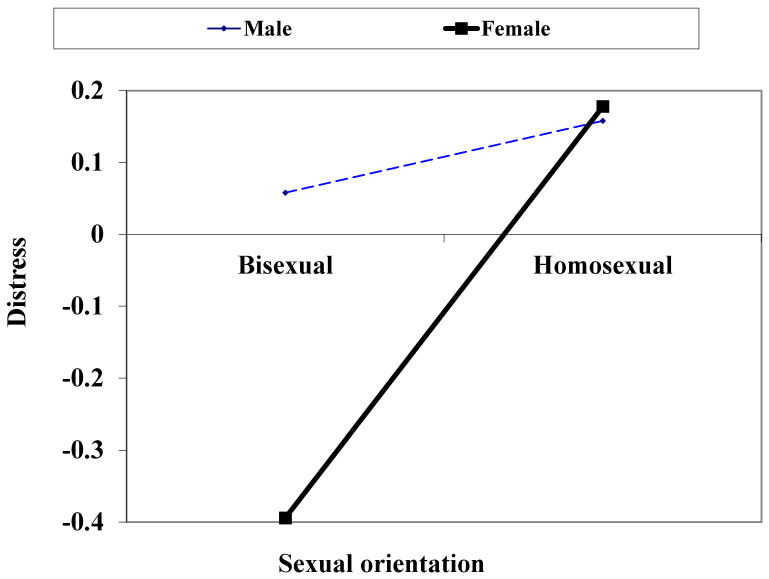
Standard score of distress by gender and sexual orientation.

In addition, significant effects from prior distress and background characteristics on distress were present. Distress in 2016 had a strong effect on distress in 2017 (*β* = 0.783, see Column (1) in Table 3). Moreover, distress was higher in the average LGB person who was homosexual rather than bisexual (*β* = 0.194, see Column (1) in Table 3), male rather than female (*β* = −0.1109), or higher in education (*β* = 0.103). Distress was additionally higher in the female homosexual person (i.e., lesbian), beyond the individual effects of gender and sexual orientation (*β* = 0.118, see Table 5). Accordingly, the average bisexual woman’s distress was considerably lower than the others (see Figure 2). By contrast, the age and location (involving Taiwan, Hong Kong, or Mainland China), rural or urban location, and social desirability did not significantly affect distress (see Table 5).

## 4. Discussion

Regarding the stress–vulnerability model, the analysis showed that the stress of social exclusion induced a significant increment in distress in the average Chinese LGB person with prior distress as a vulnerability factor. That is, social exclusion in 2016 exhibited a greater increase in distress in 2017 when distress in 2016 was higher. Meanwhile, the main effect of social exclusion on distress was not significant. Consequently, social exclusion was conditionally distressing, conditional on prior distress. Social exclusion is not generally distressing but is only distressing conditionally when prior distress is high.

The stress of social exclusion is not generally distressing, probably because the stress activates a person’s coping mechanisms to mitigate the effect of the stress. Notably, although social exclusion significantly correlated with distress, its change in distress was nonsignificant after the control for prior distress. According to the stress–vulnerability model, stress triggers a person’s coping mechanisms, and failure to cope then engenders distress [44,45]. Conversely, if coping is effective, stress will not lead to distress. At this juncture, stress has invoked coping, generally preventing distress [75,76] while, remarkably, the stress of social exclusion has generated coping [77]. According to the stress–vulnerability model, this generation happens when social exclusion is stressful enough to raise a response [44,45]. The application of the model suggests that Chinese LGB people experience social exclusion as stressful and cope with it responsively to prevent its distressing effect. This suggestion is plausible because Chinese people are generally socially concerned and can solicit social support to cope with stress and maintain social well-being [78,79].

Additionally, social exclusion is not generally distressing in the Chinese context. Specifically, this context is less likely than the Western context to regard LGB as sinful [16]. As such, social exclusion in the Chinese context is less harsh than in the West, where it is influenced by Christian or other religious disciplines [12,16]. By contrast, the Confucianism underlying the Chinese context is less religiously sanctioning than the fundamentalist religion dominant in other places [80].

The nonsignificant effect of social exclusion on the average LGB person’s distress also supports a reconsideration of the stress model [6,7]. Accordingly, prior distress rather than social exclusion significantly predicts later distress. Such a prediction illustrates the dispositional influence on distress. Another dispositional influence may stem from the average LGB person’s discounting of the impact of social norms [61]. Furthermore, the effect of social exclusion may depend on sensitivity to exclusion or rejection, which is another disposition [81]. Thus, social exclusion would not raise distress if the sensitivity to the exclusion were low.

Additionally, differences due to background characteristics are explicable with the stress–vulnerability model. In the first place, the average homosexual person had greater distress than the bisexual person, possibly because of the former’s lower flexibility when coping with stressful demands about sexuality [82]. The average bisexual person is more sexually flexible and thus more effective in avoiding social stress. This difference between a homosexual and bisexual person in distress has been notable [47]. Similarly, the average LGB woman had lower distress than the average LGB man probably because of the former’s better fulfillment of social demand and thus ability to avoid its stress [83]. The average LGB woman and the facets of her sexuality, such as monogamy and sexual flexibility, tend to fit social norms and evade social stress better than her male counterpart [84]. This is particularly the case for bisexual women, who can maintain sexual relationships with men and thus conform to the heterosexual social norm (see Figure 2). In addition, distress has been shown to be higher in those with a higher education [49]. This difference may be attributable to higher aspiration, the consequent discrepancy between their situation and reality, and thus stress [47]. A further reason in the Chinese context may be that education, conveying the orthodox cultural norm there, is less supportive of and thus more distressing to the LGB person [16].

## 5. Limitations and Future Research

The study is limited to the sampling and self-report measurement of Chinese LGB people recruited through LGB organizations based on a one-time web survey. This study cannot ensure its representativeness for Chinese LGB people as a whole, not to mention those worldwide. In addition, the one-time survey can hardly guarantee the causal inference for the effects estimated from statistical analysis. This difficulty is primarily because the survey cannot control for all of the confounding factors and ensure the temporal precedence of predictors. The self-report measurement is also imperfect because of its vulnerability to personal interpretation and expectation. What is more, the Chinese context favorable to collectivism generates social support to assuage the stressful effect of social exclusion. All of these limitations require future research in order to be addressed. Specifically, such research needs to enhance the representativeness of the LGB population, the adequacy of measurement, and the rigor of design. For representativeness, sampling needs to include Chinese LGB people as well as non-Chinese LGB people in order to optimize sociocultural diversity. This diversity allows for a moderation analysis due to sociocultural contextual factors in order to ascertain the generality and specificity of the present findings. Notably, the analysis needs to gauge the influence of the Chinese or collectivist context. Future research also needs to employ a panel and a repeated-measure design to ensure the temporal precedence of predictors and to control for the initial state of distress or any other outcome. For the measurement, triangulation with multiple informants or sources is desirable to uphold validity. Such enhancement in future research on sexual orientation issues involving LGB people can also apply to research on those with gender issues, including transgender or gender-nonconforming people.

For theoretical advancement, future research can elaborate and further develop the stress–vulnerability model, including its related stress-aggravating effect. Fundamentally, mechanisms or processes underlying the effects of social exclusion are the goals of the elaboration and development of such research. For instance, future research can elucidate the mechanism of stress aggravation by vulnerability [85,86] and, hence, focus on the way in which prior distress aggravates the distressing effect of social exclusion.

### Implications

In alleviating distress in Chinese LGB people, preventing social exclusion and its stressful effects is advisable. Notably, the prevention can target those with high distress, as social exclusion increases their distress conditionally. Similarly, Chinese LGB people who are male, homosexual, and higher in education are also targets for the prevention of social exclusion because of their higher distress. Such targeting is compatible with care ethics, which emphasizes caring for those that require it [87]. Conversely, prevention without targeting would not be as effective as many other primary preventions [88]. In general, preventing social exclusion is plausible and feasible because it is consistent with the trend of social development in the world and in the Chinese context [89,90]. Essentially, such development needs to take care of individualization and the harms that it inflicts so as to support social inclusion [91].

## 6. Conclusions

Social exclusion has raised the average Chinese LGB person’s distress conditionally, based on their prior distress. This conditional impact supports the stress–vulnerability model rather than the stress model generally. The former model is a way to refine the latter, considering the confounding of the stress effect due to dispositional or prior factors [6,7]. Such refinement warrants the targeting of LGB people suffering from distress in order to mitigate the distressing effects of social exclusion.

## Figures and Tables

**Table 1:** Percentages of sociodemographic characteristics by the site.

Characteristic	Mainland	Taiwan	Hong Kong
Age			
<30 years	81.5	31.6	43.6
30+ years	18.5	68.4	56.4
Gender			
Male	76.8	73.7	76.5
Female	23.2	26.3	23.5
Education			
High school or below	21.6	9.1	23.8
Above high school	78.4	90.9	76.2
Sexual orientation			
Bisexual	28.1	10.5	21.1
Homosexual	71.9	89.5	78.9
Residence			
Non-rural	67.9	94.7	90.1
Rural	32.1	5.3	9.9

**Table 2:** Percentages of sociodemographic characteristics by sexual orientation.

Characteristic	Bisexual	Homosexual
Age		
<30 years	69.6	52.3
30+ years	30.4	47.7
Gender		
Male	55.6	82.5
Female	44.4	17.5
Education		
High school or below	28.6	20.2
Above high school	71.4	79.8
Residence		
Non-rural	80.6	83.4
Rural	19.4	16.6

**Table 3:** Standardized factor loadings on five trait factors and one method factor.

Factor/Indicator	Trait		Method	
	2017	2016	2017	2016
Distress				
Feeling nervous	0.602	0.646	0.444	0.475
Feeling flurried	0.805	0.665	0.448	0.424
Feeling worried	0.652	0.486	0.438	0.410
Feeling troubled	0.816	0.655	0.481	0.479
(not) Calming down	0.434	0.634	−0.481	−0.535
(not) Having self-control	0.504	0.480	−0.451	−0.536
(not) Having emotional stability	0.558	0.552	−0.550	−0.527
Social exclusion experienced				
Society rejecting you		0.902		0.383
Society resisting you		0.792		0.393
Society discriminating against you		0.595		0.430
Society resenting you		0.909		0.360
Social desirability				
Being ready to help others		0.439		0.619
Treating people with disagreeable opinions courtesy		0.617		0.570
Being confident in your judgment		0.820		0.490

**Table 4:** Partial correlations.

Correlate	Distress, 2017		Distress, 2016		Social Exclusion, 2016	
Distress, 2017	1					
Distress, 2016	0.791	***	1			
Social exclusion experienced, 2016	0.149	*	0.241	***	1	
Social desirability, 2016	−0.480	***	−0.599	***	−0.264	***

Note. Controlling for acquiescence. * *p* < 0.05. *** *p* < 0.001.

**Table 5:** Standardized regression coefficients for predicting distress, 2017.

Predictor	(1)		(2)	
Hong Kong vs. Mainland	0.011		0.016	
Taiwan vs. Mainland	−0.036		−0.028	
Age	−0.059		−0.075	
Female	−0.109	**	−0.108	**
Education	0.103	**	0.096	
Homosexual vs. bisexual	0.194	***	0.168	***
Rural residence	0.037		0.042	
Social desirability, 2016	−0.081		−0.111	
Acquiescence	0.099		0.124	*
Distress, 2016	0.783	***	0.761	***
Social exclusion experienced, 2016	0.009		−0.006	
Distress × Social exclusion experienced, 2016			0.064	*
Female × Homosexual			0.118	***
*R^2^*	0.691		0.708	

Note. (1) Main effects only. (2) Interaction effects added. * *p* < 0.05. ** *p* < 0.01. *** *p* < 0.001.

## Data Availability

Data for analysis are available on reasonable request.
